# Endovascular catheter ablation of ventricular tachycardia in a patient with a surgically repaired congenital left ventricular aneurysm

**DOI:** 10.1007/s12471-015-0702-9

**Published:** 2015-05-19

**Authors:** T.T.T.K. Ramdjan, A. Yaksh, J.W. Roos-Hesselink, N.M.S. de Groot

**Affiliations:** Department of Cardiology, Thoraxcentrum, Erasmus MC, Rotterdam, The Netherlands

**Keywords:** Congenital left ventricular aneurysm, Ventricular tachycardia, Catheter ablation

## Abstract

We present a patient with a congenital left ventricular aneurysm who visited our outpatient clinic for a routine check-up and, during this visit, lost consciousness due to sustained ventricular tachycardia. In our patient, endocardial mapping revealed extensive conduction abnormalities, and successful ablation was accomplished at the endocardial surface.

A 19-year-old male individual with a surgically corrected congenital left ventricular aneurysm (LVA) in the basal posterior wall visited our outpatient clinic for a routine check-up. Surgical resection of the aneurysm was performed 10 years ago. LVA recurrence was discovered only 1 year after surgery. The left ventricle was dilated (end-diastolic diameter 70 mm), and the ejection fraction was decreased (36 %). During this visit, the patient lost consciousness due to sustained ventricular tachycardia (VT; left upper part of Fig. 1). His ICD (implantable cardiac defibrillator), which was implanted earlier for non-sustained VT episodes, did not deliver therapy, as the cycle length of the VT was longer than the programmed detection interval. The VT seemed to be originating from the basal posterior wall of the left ventricle, most likely from the LVA. During external cardioversion, the VT degenerated into ventricular fibrillation. Sinus rhythm was finally achieved after external defibrillation combined with amiodarone infusion. A computed tomography scan, performed prior to the ablation procedure, clearly showed an aneurysm in the basal posterior wall of the left ventricle (central part of Fig. 1). Subsequently, the patient was scheduled for ablative therapy of the VT. The mapping/ablation procedure was guided by a three-dimensional electro-anatomical mapping system (Ensite NavX™, St, Jude Medical, St. Paul, MN, USA). First, an anatomical reconstruction was created during sinus rhythm to localise the LVA; electrograms with an abnormal morphology (low voltage, double and fractionated potentials) were recorded from this area. After accurate delineation of the LVA (white circle in Fig. 1), a VT with a cycle length of 290 ms, which was identical to the clinical VT, was induced by fixed rate pacing (cycle length: 270 ms) and could repeatedly be terminated by overdrive pacing (cycle length: 210 ms). The earliest activation relative to the QRS complex during VT was found within the aneurysm. Pacing at this site resulted in entrainment with concealed fusion and a post-pacing interval of 10 ms (left lower part of Fig. 1). The VT terminated during the first radiofrequency application. The clinical VT was non-inducible after encircling the site of earliest activation. After the ablation procedure, only a non-clinical VT originating from the basal septum of the left ventricle could be induced with aggressive stimulation (cycle length: 210 ms), which resulted in haemodynamic instability and was terminated by overdrive pacing. The surface electrocardiograms before and after the ablation procedure are demonstrated in Fig. 2. One year after the ablation procedure, the ICD print showed one asymptomatic episode of non-sustained VT. Sustained VT, however, did not recur again.

**Fig.1:**
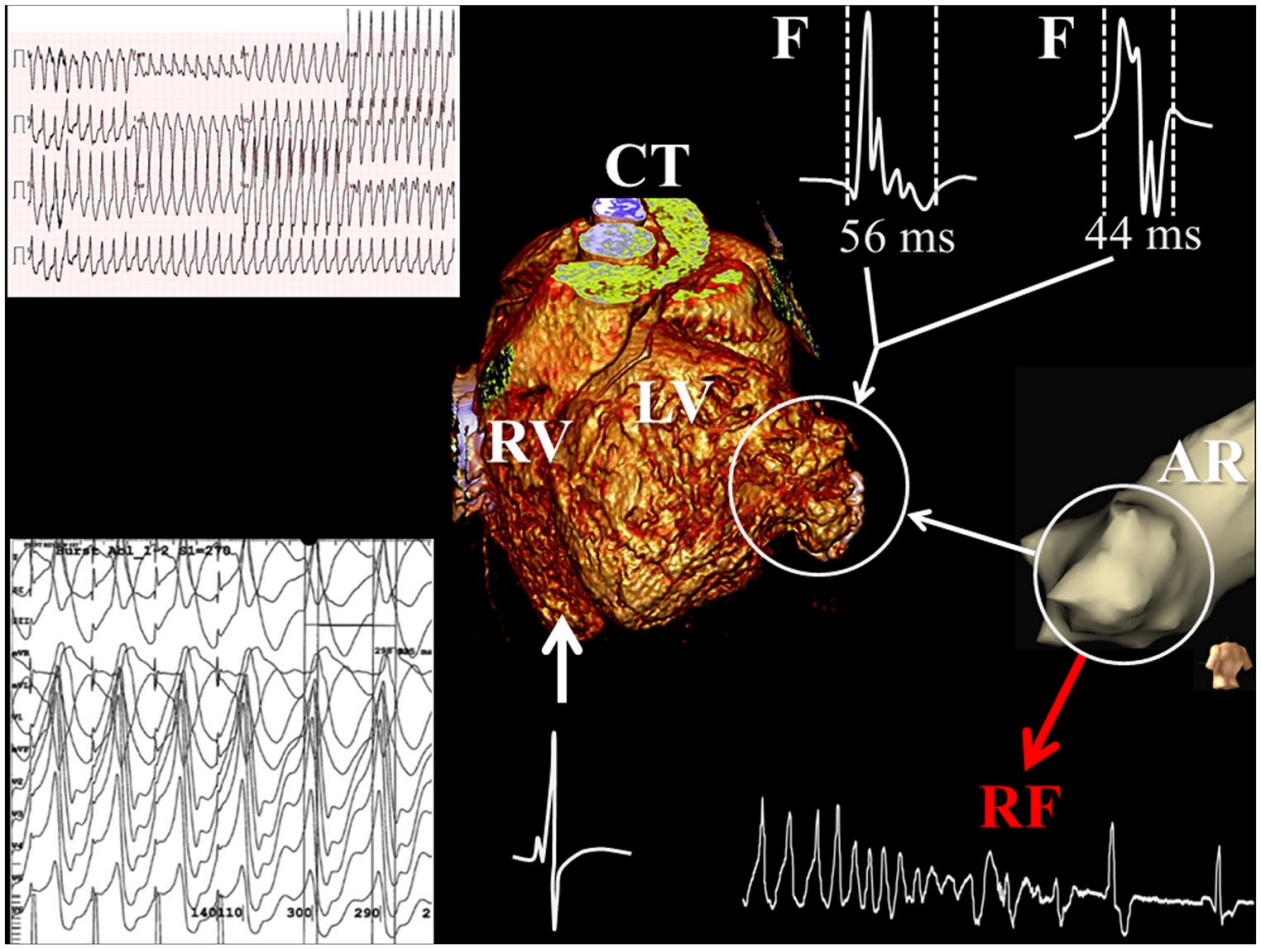
Electro-anatomical mapping of ventricular tachycardia. Surface electrocardiogram demonstrating a monomorphic ventricular tachycardia (VT) of 218 beats/min. The computed tomography (*CT*) scan showed an aneurysm in the basal posterior wall of the left ventricle (*white circle*). During the mapping procedure, an anatomical reconstruction (*AR*) was created during sinus rhythm to accurately delineate the left ventricular aneurysm (LVA). Fractionated electrograms (*F*) were observed during sinus rhythm within the LVA, indicating local dissociation in conduction. During VT, pacing within the LVA area resulted in entrainment with concealed fusion (*left lower panel*). Radiofrequency (*RF*) ablation at this site terminated the VT

**Fig. 2:**
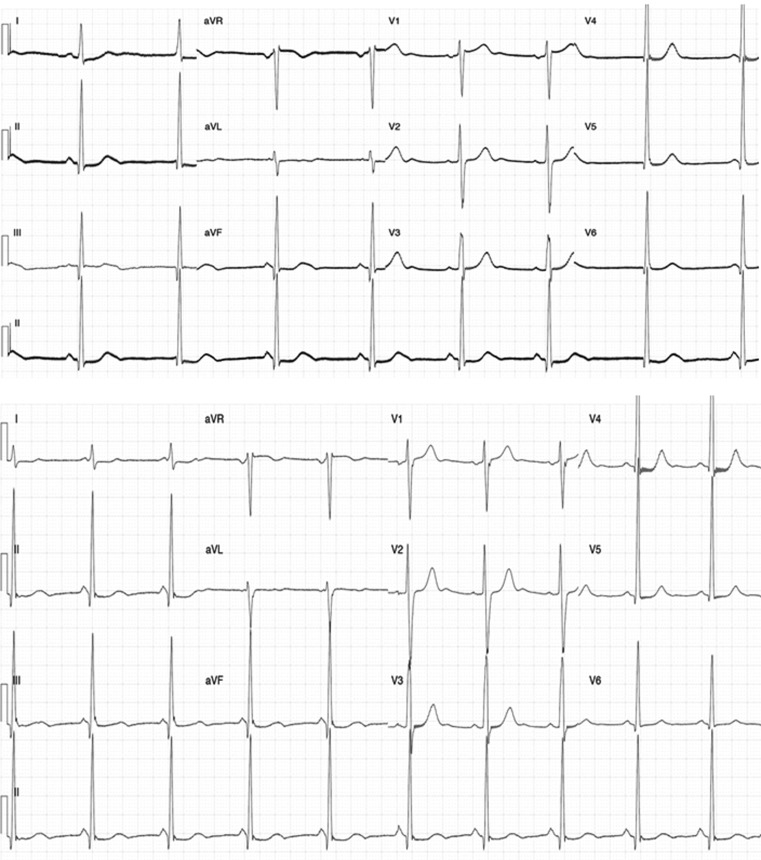
Surface electrocardiogram (ECG) of sinus rhythm before and after the ablation procedure. Surface ECG obtained just before (*upper panel*) and 1 day after (*lower panel*) the ablation procedure

## Discussion

Congenital LVA is a rare congenital defect. It is defined as the presence of an akinetic or dyskinetic structure with a wide connection to the left ventricle, which is normal in size and function [1]. Reports on VT associated with congenital LVA are limited to a few cases [2]. Patients with LVA are frequently asymptomatic but congestive heart failure, tachyarrhythmias and systemic embolisation may develop. Even sudden death has been observed in LVA patients and may be related to rupture of the LVA [3, 4].

In a group of 108 patients with either LVA or diverticulum, who were followed during a median period of 50 months, VTs occurred in only ten patients [5]. Yamashiro et al. [6] described cryoablation during cardiac surgery in two adult LVA patients with sustained VT. However, they did not perform epicardial mapping prior to ablation. Only Ouyang et al. reported on cardiac mapping prior to ablation in four LVA patients with recurrent exercise-induced syncope attributable to sustained fast monomorphic VT. The aneurysm was located in the inferolateral part of the left ventricle in all patients. Mapping during sinus rhythm revealed abnormal signals (fractionated or late potentials) on the epicardium but not on the endocardium. In one patient with a stable VT, epicardial mapping showed a macrore-entrant VT with a focal pattern of activation at the endocardial surface. Epicardial ablation was successfully performed in three patients [7]. Hence, our case is the first LVA patient in whom (1) endocardial mapping revealed extensive conduction abnormalities (low voltage, double and fractionated potentials), and (2) successful ablation was accomplished at the endocardial surface. Our patient had a dilated left ventricle and depressed ejection fractionindicating a more advanced stage of LVA compared with the patients in the study by Ouyang et al. [7]. The underlying mechanism of the VT is most likely a microre-entry circuit at the endocardial surface. However, endocardial breakthrough of an epicardial wavefront cannot be excluded.

## Conclusion

Ventricular tachycardia after cardiac surgery for LVAs can be treated by endocardial catheter ablation at the endocardial surface of the left ventricle. Extensive local conduction abnormalities are recorded within the endocardial surface of the aneurysm which may form the substrate of the ventricular tachycardia.

### Funding

None.

### Conflict of interests

None declared.
